# Evaluation of novel inducible promoter/repressor systems for recombinant protein expression in *Lactobacillus plantarum*

**DOI:** 10.1186/s12934-016-0448-0

**Published:** 2016-03-10

**Authors:** Silvia Heiss, Angelika Hörmann, Christopher Tauer, Margot Sonnleitner, Esther Egger, Reingard Grabherr, Stefan Heinl

**Affiliations:** Christian Doppler Laboratory for Genetically Engineered Lactic Acid Bacteria, Department of Biotechnology, University of Natural Resources and Life Sciences, Muthgasse 11, 1190 Vienna, Austria

**Keywords:** *L. plantarum* 3NSH, BioLector^®^ micro-fermentation system, Orthologous expression system, T7 RNA polymerase, IPTG, Inducible expression

## Abstract

**Background:**

Engineering lactic acid bacteria (LAB) is of growing importance for food and feed industry as well as for in vivo vaccination or the production of recombinant proteins in food grade organisms. Often, expression of a transgene is only desired at a certain time point or period, e.g. to minimize the metabolic burden for the host cell or to control the expression time span. For this purpose, inducible expression systems are preferred, though cost and availability of the inducing agent must be feasible. We selected the plasmid free strain *Lactobacillus plantarum* 3NSH for testing and characterization of novel inducible promoters/repressor systems. Their feasibility in recombinant protein production was evaluated. Expression of the reporter protein mCherry was monitored with the BioLector^®^ micro-fermentation system.

**Results:**

Reporter gene mCherry expression was compared under the control of different promoter/repressor systems: P_lacA_ (an endogenous promoter/repressor system derived from *L. plantarum* 3NSH), P_xylA_ (a promoter/repressor system derived from *Bacillus megaterium* DSMZ 319) and P_lacSynth_ (synthetic promoter and codon-optimized repressor gene based on the *Escherichia colilac* operon). We observed that P_lacA_ was inducible solely by lactose, but not by non-metabolizable allolactose analoga. P_xylA_ was inducible by xylose, yet showed basal expression under non-induced conditions. Growth on galactose (as compared to exponential growth phase on glucose) reduced basal mCherry expression at non-induced conditions. P_lacSynth_ was inducible with TMG (methyl β-D-thiogalactopyranoside) and IPTG (isopropyl β-D-1-thiogalactopyranoside), but also showed basal expression without inducer. The promoter P_lacSynth_ was used for establishment of a dual plasmid expression system, based on T7 RNA polymerase driven expression in *L. plantarum*. Comparative Western blot supported BioLector^®^ micro-fermentation measurements. Conclusively, overall expression levels were moderate (compared to a constitutive promoter).

**Conclusions:**

We evaluated different inducible promoters, as well as an orthologous expression system, for controlled gene expression in *L. plantarum*. Furthermore, here we provide proof of concept for a T7 RNA polymerase based expression system for *L. plantarum*. Thereby we expanded the molecular toolbox for an industrial relevant and generally regarded as safe (GRAS) strain.

**Electronic supplementary material:**

The online version of this article (doi:10.1186/s12934-016-0448-0) contains supplementary material, which is available to authorized users.

## Background

*Lactobacillus plantarum* is a versatile lactic acid bacterium that is generally regarded as safe (GRAS). It inhabits diverse ecological niches and exhibits probiotic characteristics [42]. *L. plantarum* is often used as starter or adjunct culture in fermented food and feed production processes like for sausages, cheeses, fermented vegetables, and grass or corn silage [10, 36, 37]. Due to its high oxygen tolerance and robustness in natural fermentation processes, *L. plantarum* has gained increasing interest also as a host for recombinant protein expression and thus, its use in biotechnological applications is steadily growing [1, 20, 41]. Research involves genomics, transcriptomics, cell engineering and evolutionary strain optimization [9, 37] e.g. for bulk production of chemicals, metabolites and enzymes [23, 28] as well as for in situ delivery of vaccines [8, 11, 12, 32, 50]. Anti-microbial features, such as plantaricin production, are also of growing importance [33].

Specific gene regulatory elements like promoters are a prerequisite for efficient transcription of recombinant genes in any host organism. Accordingly, several constitutive promoters and shuttle vector systems have been established [38, 43, 44, 47]. Often, constitutive expression is preferred, for example for in situ delivery of recombinant proteins in the human body, or when steady-state gene expression is required [38]. Contrarily, constitutive promoters do not allow regulation of gene expression and production levels are directly linked to cellular growth. Continuous transcription throughout the fermentation process poses a limit to the expression of foreign proteins, which are potentially toxic to the host cell or exhibit excessive metabolic burden.

An alternative strategy is to use substrate dependent promoters that can be induced after a certain cell density has been reached. Several inducible promoters for *L. plantarum* have been described in the literature. The nisin-controlled gene expression (*NICE*) system is inducible with the bacteriocin nisin from *Lactococcus lactis* and was established also for *L. plantarum* [25]. However, the expression is not tightly regulated except if the target expression cassette is integrated into the host’s chromosome [34]. The pSIP system comprises a well-established inducible promoter system and is based on the induction of promoters from *Lactobacillus sakei* with an inducing peptide [46]. More recently, another inducible promoter based on manganese starvation was described for *L. plantarum* NC8 [3].

Yet, numerous other substrate induced promoter-repressor systems are present in LAB and other bacteria that eventually may serve to efficiently control transgene expression. *Lactobacillus plantarum* contains a *lac* - operon which was expected to be regulated similarly as the well-studied *lac*-operon of *Escherichia coli*, where the *lac*-operon comprises the genes *lacZ* (β-galactosidase), *lacY* (lactose permease), *lacA* (transacetylase) and *lacI* (repressor). Allolactose is the natural inducer of the *lac*-operon. In *E. coli*, thio-galactosides such as IPTG (isopropyl β-D-1-thiogalactopyranoside) and TMG (methyl β-D-thiogalactopyranoside) are the most commonly used inducers in industrial production processes.

We established a synthetic inducible promoter system based on the *E. coli* derived *lac*-operon, which we adapted for *L. plantarum* in the high copy number shuttle vector pCDLbu1 [15, 43]. Based on the inducible synthetic system, we designed and constructed an artificial T7 RNA polymerase regulated dual plasmid expression system and demonstrated its applicability in *L. plantarum* 3NSH. Additionally, we tested endogenous *lac*-operon regulatory sequences from *L. plantarum* 3NSH. This strain is derived from *L. plantarum* CD033, which was cured of its native plasmid [17]. Plasmid free strains are preferable expression hosts, since native plasmids sometimes interfere with expression vector replication.

Another well-known regulated system is the xylose operon and the xylose promoter/repressor gene from *Bacillus megaterium*, which is well established for Gram-positive bacteria, and was already used for high yield production of secretory levansucrase in *B. megaterium* YYBm1 [22] and dextransucrase in *B. megaterium* MS941 [26]. Moreover, three different recombinant proteins in *Brevibacillus choshinensis* SP3 under the control of P_xylA_ from *B. megaterium* have been reported [5]. D-xylose is metabolized by two intracellular enzymes: the D-xylose isomerase (XylA) and the D-xylose kinase (XylB). D-xylose can be transported into the cell by two different mechanisms. One mechanism involves a D-xylose-H^+^ or –Na^+^ symporter (*xylT*) and is regulated by CcpA [40]. Another mechanism is driven by ATP and consists of a high-affinity xylose transporter system involving a periplasmic binding protein. For three species of facultative hetero-fermentative lactobacilli, *Lactobacillus pentosus*, *L. plantarum*, and *Lactobacillus casei* it was shown that EII^Man^ complex of the phosphoenolpyruvate (PEP): D-mannose phosphotransferase system (PTS) is involved in D-xylose transport via facilitated diffusion [4]. Posno and co-workers [35] reported that *L. plantarum* does not metabolize D-xylose. For its use as an inducer, this is an advantage, since D-xylose is not degraded and keeps the level of induction constant throughout the process.

In this study, we present the establishment and characterization of different inducible promoter/repressor systems (and their respective inducer) in the high copy number pCDLbu1 shuttle vector for *L. plantarum* 3NSH. We used mCherry as reporter protein and expression levels were analyzed with the BioLector^®^ micro-fermentation system and confirmed by Western blot immuno-detection. Furthermore, we established an inducible T7 RNA polymerase based system for regulated recombinant gene expression. Summarizing, we present expression plasmids with a set of novel inducible promoters, and expand the toolbox for recombinant protein expression in *L. plantarum*.

## Results and discussion

Different inducible promoter systems were characterized and tested in the plasmid free strain *L. plantarum* 3NSH [17]. Comparative studies were carried out regarding bacterial growth rates, level of reporter gene expression, effect of inducer and behavioral differences due to varying carbon sources. BioLector^®^ micro-fermentations were established. FlowerPlates (with or without optodes for low pH and dissolved oxygen) for detection of biomass (calculated optical density) and fluorescence were used for *L. plantarum* fermentation and analysis.

**Fig. 1:**
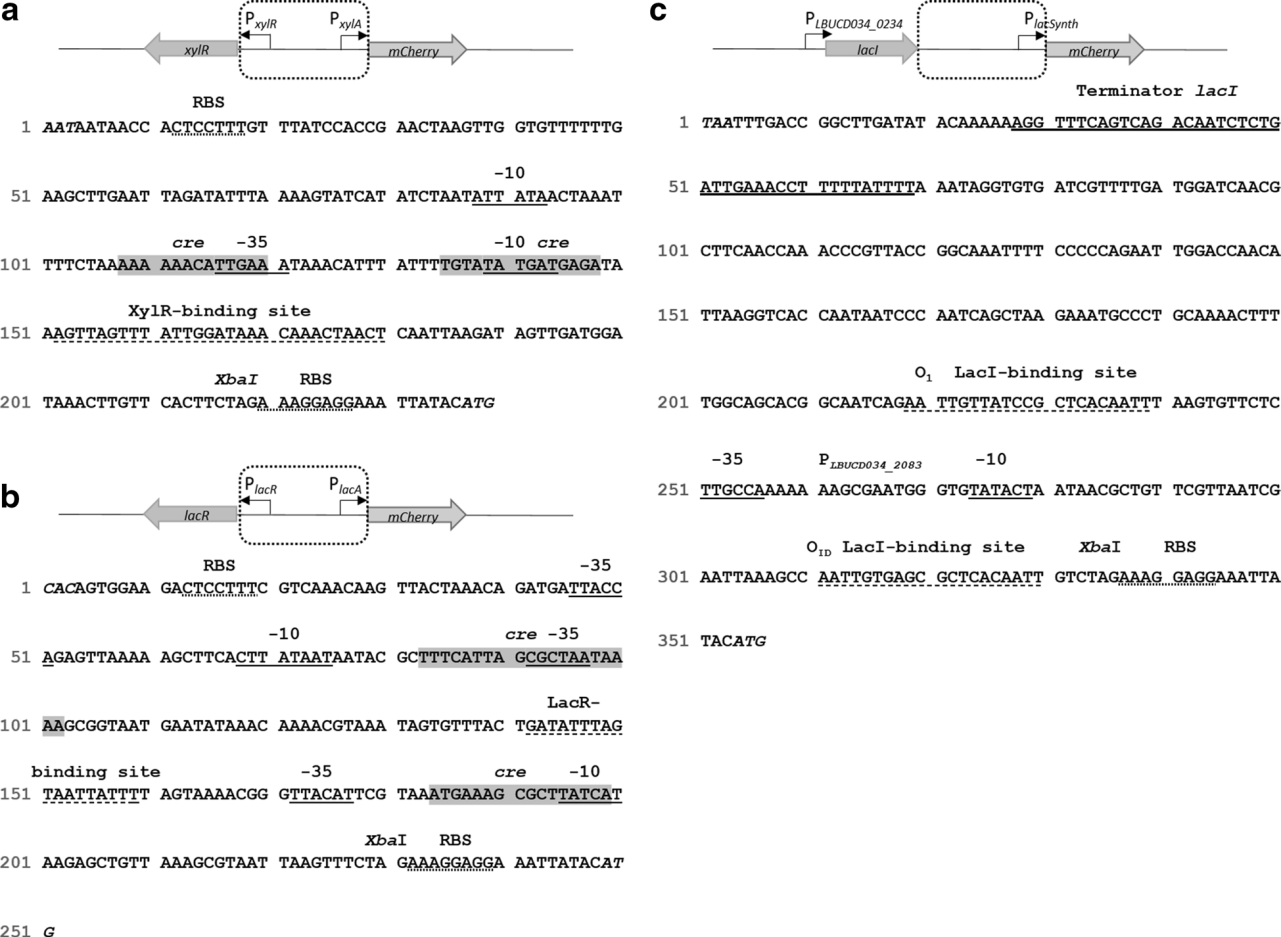
Promoter region sequences of P_xylA_, P_lacA_ and P_lacSynth_. Nucleotide sequence from repressor to start codon of mCherry are shown (region within *dotted square*). The mCherry start codon is indicated in *italics*, preceded by an identical ribosome binding site (*RBS; italics*) an *Xba*I restriction site (*bold*) and an identical 9 nt spacer sequence was introduced upstream of mCherry start codon. The −35 and −10 promoter region were identified (SoftBerry, BPROM) and are *underlined*. Primer binding sites for negative controls (for construction of negative controls without promoter) are underlined in *dashed line*. **a** P_xylA_; promoter of *xylA* gene from *B. megaterium* DSMZ 319 and promoter of repressor XylR. Operator sequences for XylR binding are underlined; *cre* sites (catabolite-responsive element) are *highlighted*. **b** P_lacA_; endogenous promoter of LacA from *L. plantarum* 3NSH and promoter of repressor LacR. LacR-binding site was identified (RegPrecise) and *underlined*, and putative *cre*-sites are highlighted. **c** P_lacSynth_; promoter P_2083_ from *L. buchneri* CD034 with artificially integrated operator binding sites with recommended distance of 93 nt (O_1_ and O_iD_ from *E. coli*) are *underlined* (*dotted line*), terminator of *lacI* is *underlined* (*solid line*)

Besides promoter elements and transcription initiation, other factors additionally influence the level of protein expression. Such are terminators, untranslated regions, plasmid copy numbers and the protein itself (amino acids, folding, and toxicity). In our experimental set-up, we chose the ribosomal binding site (RBS) and the spacer between the RBS and start codon to be identical for all promoter constructs (Fig. 1), in order to exclude any translational effects on mCherry expression caused by different RBSs. However, the promoter consensus sequences (including the −35 and −10 region) were specific for each tested promoter.

### The heterologous promoter P_xylA_ is inducible by xylose

The promoter fragment P_xylA_ and the repressor gene cassette XylR were amplified from *B. megaterium* DSMZ 319 genomic DNA with primers listed in Table 1. Nucleotide sequences of promoters P_xylA_ and promoter P_xylR_ to start codon of mCherry are shown in Fig. 1a, where promoter, RBS, *cre* (catabolite-responsive element)-sites and XylR binding site are indicated. The xyl-repressor binding motif is indicated according to Stammen and colleagues [45].

**Table 1:** List of primers used in this study

Name	5´–3´ Sequence
PlacSynth_*Sac*I_*Eco*RI_F	GATGAC**GAGCTC** *GAATTC*TGGTCTTTATTCTTCAA
M13_R_*Nhe*I	CGACGA**GCTAGC**AGCCAGGAAACAGCTATGACC
mCherry_RBS_*Xba*I	GCTGCT**TCTAGA**AAGGAGGAAATTATACATGTTATCAAAGGGTGAAGAAG
mCherry_R_*Bam*HI	CGTCGT**GGATCC**TTATCACTTGTATAATTCATCCATACC
Tldh_amp_R_*Pst*I	CTGCTG**CTGCAG**AAAAAGATTAAAAAAGCCGCTGC
mCherry_seq_R	TGGACGACCTTCACCTTCAC
mCherry_seq_F	AACGTATGTACCCAGAAGATG
CAT_seq2_back	TACATCATTCTGTTTGTGATGG
B_mega_XylOP_out_F	AACATATAAACAGCCAGTTGCC
B_mega_XylOP_R(*Spe*I, *Sca*I, *Bam*HI)	GTAGTA**GGATCC** *AGT* ***ACTAGT***TTCCCCCTTTGATTTAAGTG
mCherry_w/o_RBS_*Xba*I	CGTCGT**TCTAGA**ATGTTATCAAAGGGTGAAGAAGATAAC
p256_miniori_for	CATCATAAGCTTCCCGCACGCATAGCGGTGC
B_mega_XylOP_F_*Mfe*I, *Kpn*I	GTAGTA**CAATTG** *GGTACC*AAGGTGAGGGTGGAGACAG
Bmega_XylR_newRBS_*Xba*I_Phos_R	GTATAATTTCCTCCTT**TCTAGA**AGTGAACAAGTTTATCCAT
mCherry_Phos_F	ATGTTATCAAAGGGTGAAGAAG
B_mega_XylOP_seq_F	CAATTCCGATATTAATACTGATG
B_mega_XylOP_seq_R	CTAGTCGGAATAGGAATTTGTG
LacI_Lplant_F_*Sac*I	AGCAGC**GAGCTC**CCTAATAGAACTGCGGTGGTC
LacI_Lplant_R_*Xba*I	AGCAGC**TCTAGA**AACTTAATTACGCTTTAACAGC
lacR_Gal_seq_R	AATTGAAGTGATGCGGGTCTG
lacR_Gal_seq_F	AATTGCGCCAGCTAACACCC
T7_RNAP_Lp_RBS	CAGCAGTCTAGATCCTAAAGGAGG
T7_RNAP_Lp_Term_R_*Sal*I	CAGCAG**GTCGAC**TTGATATACAAAAAAGG
M13_2_F	TTGTAAAACGACGGCCAGTG
T7-Promoter_*Sac*I	GCTGCT**GAGCTC**AGATCGATCTCGATCCCGCG
T7-Terminator_*Sal*I	GCTGCT**GTCGAC**TCCGGATATAGTTCCTCCTTTC
ery_back_*Kas*I	CATCAT**GGCGCC**TCCGATTGCAGTATAAATTTAACG
oripE194_seq_back	AATCAAATCGGTATAAATCTGAC
Ery_F_*Nhe*I	CATCAT**GCTAGC**TCCGATTGCAGTATAAATTTAACG
Pempty_*Sac*I_R	TAGTAG**TCTA** ***GA*** *GCTC*GAATTCACTGGCCGTCG
mCherry_RBS_*Sac*I_F	GCTGCTGAGCTCAAGGAGGAAATTATACATGTTATCAAAGGGTGAAGAAG

Restriction sites are underlined and highlighted in bold or italics

**Fig. 2:**
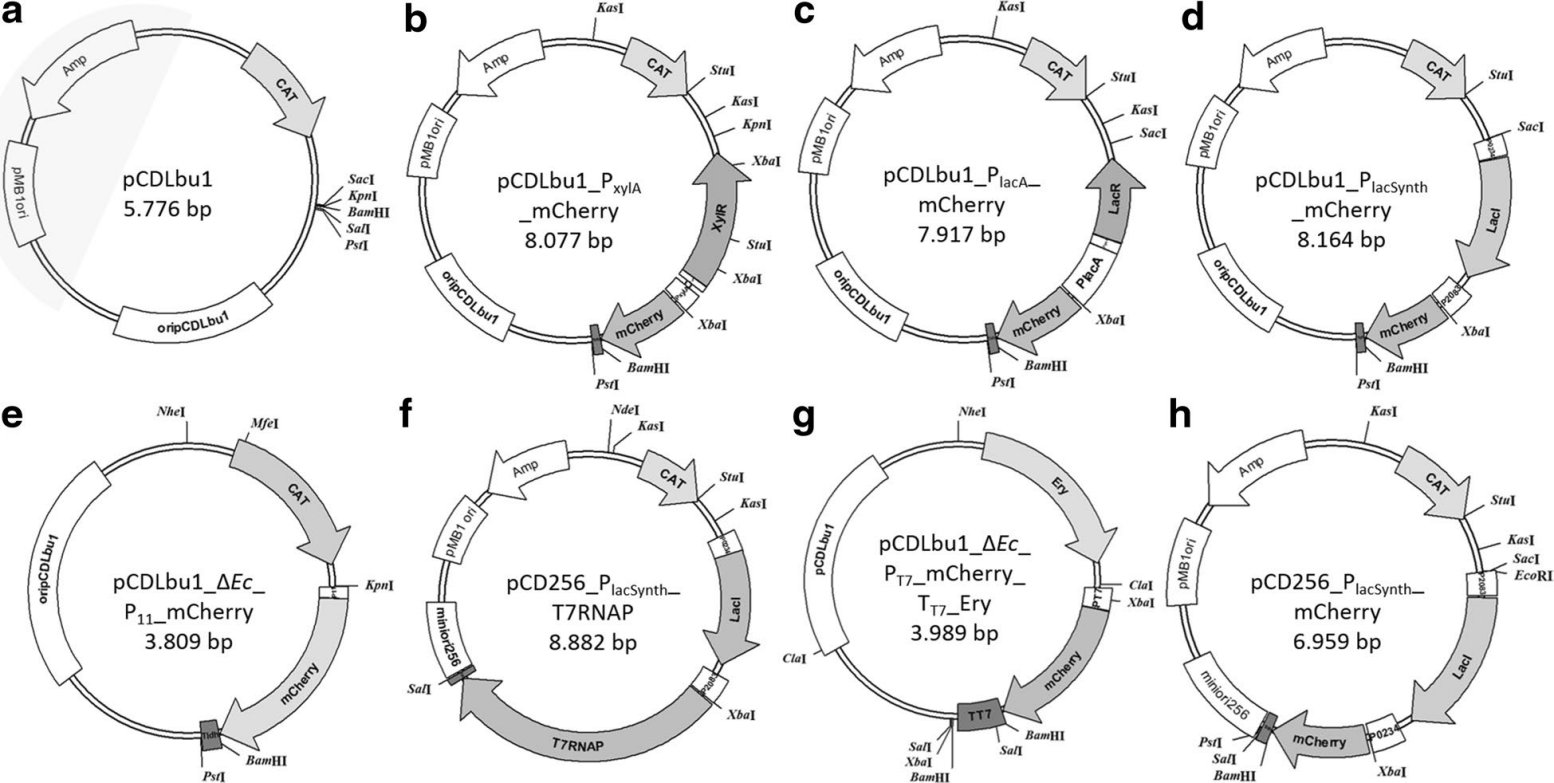
Maps of plasmids used in this study. Annotations and relevant restriction sites are indicated. **a** pCDLbu1 initial vector backbone (highlighted region are *E. coli* specific sequences); **b** pCDblu1_P_xylA__mCherry; **c** pCDLbu1_P_lacA__mCherry; **d** pCDLbu1_P_lacSynth__mCherry; **e** pCDLbu1Δ*Ec*_P_11__mCherry (constitutive P_11_ promoter; internal reference plasmid described by Tauer and colleagues [47]); The term ‘Δ*Ec’* indicates removal of *E. coli* specific sequences which are highlighted in plasmid A. **f** pCD256_P_lacSynth__mCherry; **g** pCD256_P_lacSynth__T7RNAP; **h** pCDLbu1Δ*Ec*_P_T7__mCherry _T_T7__Ery. OripCDLbu1 and miniori256: origins of replication (ori) for *L. plantarum* 3NSH; pMB1ori: ori for replication in *E. coli*; *CAT* chloramphenicol acetyltransferase gene; *Amp* ampicillin resistance gene; *Ery* *ermI* gene encoding resistance to erythromycin; *P* promoter; *T* terminator of transcription. Subscripted characters are specifications. Important restriction sites are indicated

Description of the operon and its regulation was presented by Schmiedel and colleagues [40]. Preliminary tests were performed with native RBS from XylA from *B. megaterium* DSMZ 319 in pCDLbu1_P_xylA(nativeRBS)__mCherry. The comparison of RBS and spacer sequence of P_xylA (native RBS)_ and P_xylA_ is shown in Additional file 1: Figure S1. We compared mCherry expression with native RBS to the uniform and artificial SOPT#9 spacer RBS and sequence (5´-*TCTAGA*AAGGAGGAAATTATAC**ATG**-3´, from *Xba*I to start codon), which was established for *L. plantarum* CD033 [47]. SOPT#9 was used for all constructs and allowed comparison of mCherry expression apart from translational influences. Interestingly, we found that SOPT#9 lead to slightly higher expression levels compared to the native *xyl*A RBS and spacer sequence (data not shown) and was well suited for recombinant protein expression in *L. plantarum* 3NSH. Parental rolling circle replicating plasmid pCDLbu1 is shown in Fig. 2a. The final shuttle vector pCDLbu1_P_xylA__mCherry for P_xylA_ regulated mCherry expression is depicted in Fig. 2b.

**Fig. 3:**
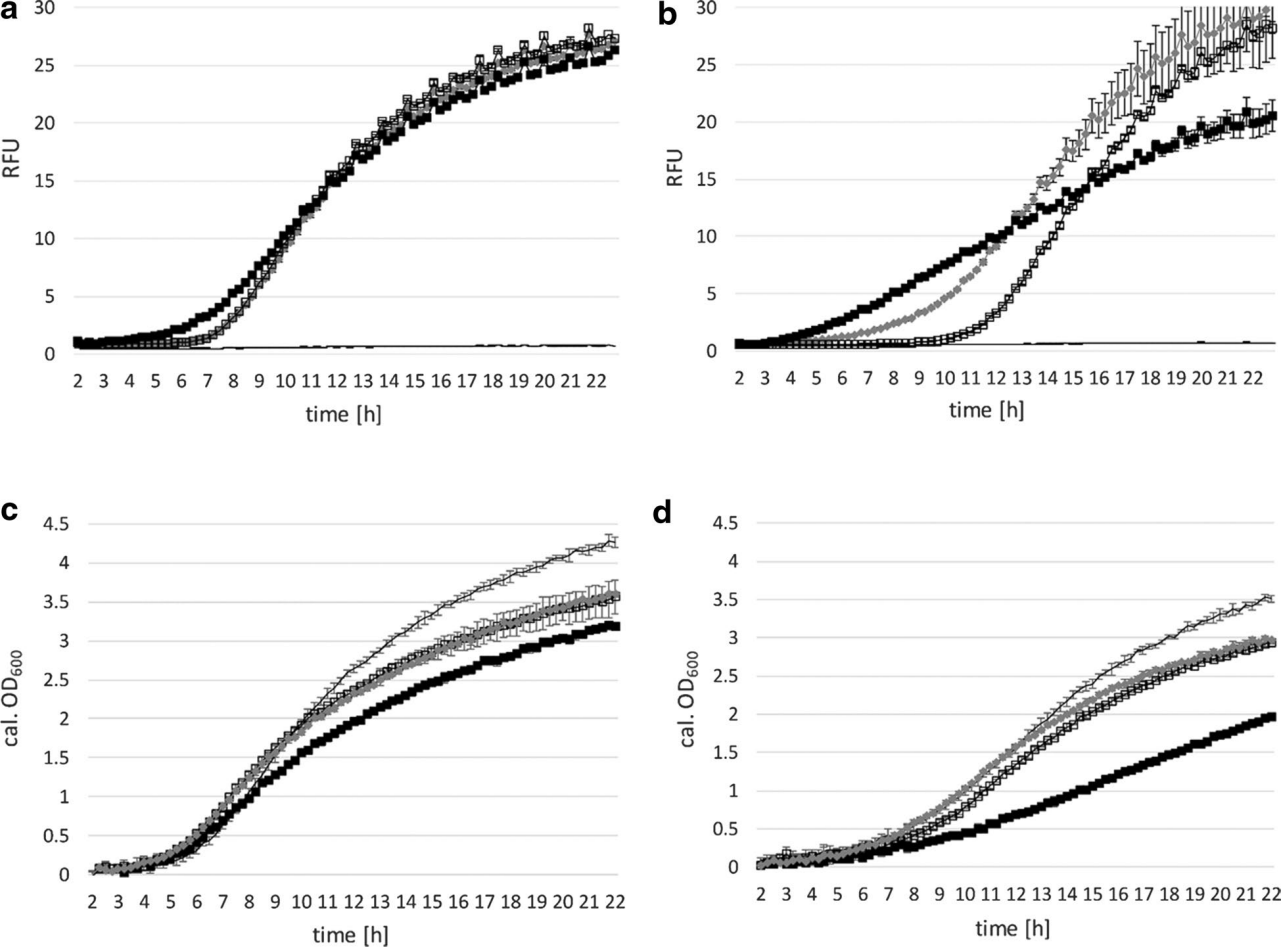
Promoter characteristics of P_xylA_ and growth behavior. **a**, **c** MRS medium with glucose and 5 μg ml^−1^ CM; **b**, **d** MRS medium with galactose and 5 μg ml^−1^ CM, mean values of four replicates are given and standard deviations are indicated. *Filled square* induced 2 % xylose. *Filled diamond* induced 0.2 % xylose. *Square* non-induced. *Solid line* negative control. **a**,**b** specific expression levels of mCherry under control of P_xylA_ after induction with 2, 0.2 % xylose (or no induction) after 2 h in BioLector^®^ micro-fermentation. Change of mCherry expression (RFUs, relative fluorescent units) over time (hours) of P_xylA_ mediated expression in comparison to the negative control is shown. **c** and **d** corresponding calculated OD_600_ values

Cells were grown on selective media with either glucose (Fig. 3a, c) or galactose (Fig. 3b, d) as main carbon source, induced with xylose (or absence of inducer) after 2 h and analyzed. Figure 3a, b show relative fluorescence units (RFUs) of mCherry expression (with or without induction) under control of P_xylA_ for 23 h. In related literature the used amount of xylose added as inducer varies from 0.5 % (*w*/*v*) in *Bacillus megaterium* to 0.2 and 2 % in *B. subtilis* [2, 22, 26]. Figure 3a shows that the addition of 0.2 or 2 % xylose in MRS medium with glucose as main carbon source showed no effect on mCherry expression as compared to non-induced conditions. Figure 3b indicates that growth on galactose and induction with 0.2 or 2 % xylose led to enhanced expression of mCherry expression during exponential growth phase. Moreover, basal expression in medium containing 2 % galactose as main carbon source was repressed during the exponential phase (Fig. 3b), as compared to growth on glucose (Fig. 3a). *Lactobacillus plantarum* 3NSH is incapable of metabolizing xylose (data not shown), but xylose is efficiently transported into the cell. The use of this promoter/repressor-system in lactobacilli was tested here for the first time.

Additionally, we tested a negative control (expression plasmid without the promoter/repressor fragment), which did not show any mCherry expression (Fig. 3a, b, solid line). Thus, basal expression was caused by weak repression of P_xylA_ through inefficient XylR repressor binding and not by any putative additional regulatory sequences present on the plasmid.

In *B. megaterium*, the presence of glucose was shown to cause repression of P_xylA_ by CcpA (catabolite control protein A) binding *cre*-sites within the promoter region and the *xylA* gene [13]. The *xylA* promoter in our context (Fig. 1a) contains two *cre*-sites, which were termed cre*-35.5 and (*cre*)-8.5 (a *cre*-like site) by Gösseringer and coworkers [13] who also showed that in *B. megaterium* the *cre* + 130.5 (within *xylA* sequence) and cre*-35.5 are simultaneously bound by CcpA, which results in looping of intervening DNA and tight repression of *xylA* transcription. Interestingly, we did not observe catabolite repression of mCherry expression by the presence of glucose with our construct (Fig. 3a). A reason for this could be the lack of *cre* + 130.5 within the mCherry gene, hence, multimer formation and efficient catabolite repression is prevented. Another explanation for the relatively strong basal expression level could be that glucose inhibits DNA binding by XylR and acts as a low-efficiency inducer for XylR as reported by Dahl and co-workers [6]: similar structure of xylose and glucose enable both sugars to utilize the same binding site on repressor XylR.

Plasmid pCDLbu1_P_xylA__mCherry containing cells only showed minor growth differences on selective medium with either glucose or galactose (Fig. 3c, d). Growth on galactose slightly increased mCherry expression and decreased basal expression levels, resulting in an improved regulation of the system during exponential phase (Fig. 3b). We hypothesize that galactose interferes less with XylR mediated repression in *L. plantarum* than glucose and, hence, leads to improved repression of mCherry expression.

*Lactobacillus plantarum* 3NSH does not metabolize xylose, but effective transportation of xylose was demonstrated through inducibility of expression. The *L. plantarum* WCFS1 complete genome sequence [21] suggests genes involved in transport (lp_0331, lp_0975), but no *xylA* or *xylB*. Chaillou et al. [4] report that EII^Man^ complex of the phosphoenolpyruvate (PEP): D-mannose PTS is involved in D-xylose transport via facilitated diffusion. For industrial processes, it is considered an advantage, when the inducing substance is not degraded and a constant concentration during cultivation can be maintained. In terms of plant based biomass degradation, where xylose is highly abundant, this expression regime could provide a self-inducing promoter system for the production of e.g. endoglucanases and xylanases, thereby increasing the rate and efficacy of substrate metabolism in ensiling processes.

### The endogenous promoter P_lacA_ is inducible by lactose

The promoter of *lacA* (β-galactosidase) and the promoter of the Lac repressor (*lacR*) were amplified from *L. plantarum* 3NSH genomic DNA with primers shown in Table 1. Figure 1b shows the nucleotide sequences of endogenous promoters P_lacA_ and promoter P_lacR_ in divergent orientation. LacR binding site, *cre*-site and RBS are indicated. The final shuttle vector pCDLbu1_P_lacA__mCherry is shown in Fig. 2c.

For promoter characterization, mCherry expression under induced and non-induced conditions was monitored. Lactose as well as the non-metabolizable lactose analogues isopropyl-β-D-thiogalactopyranoside (IPTG) and thiomethyl-β-D-galactoside (TMG) were tested for induction of P_lacA_. IPTG and TMG failed to induce LacR controlled gene expression (data not shown). This is in contrast to previous findings, where TMG was successfully used for the induction of ß-galactosidase expression in *L. plantarum* ATCC^®^ 8014™ [19]. Different sugars were tested for induction of P_lacA_ (including lactose, xylose, fructose, glucose, maltose, arabinose and galactose), but P_lacA_ was only induced with lactose.

**Fig. 4:**
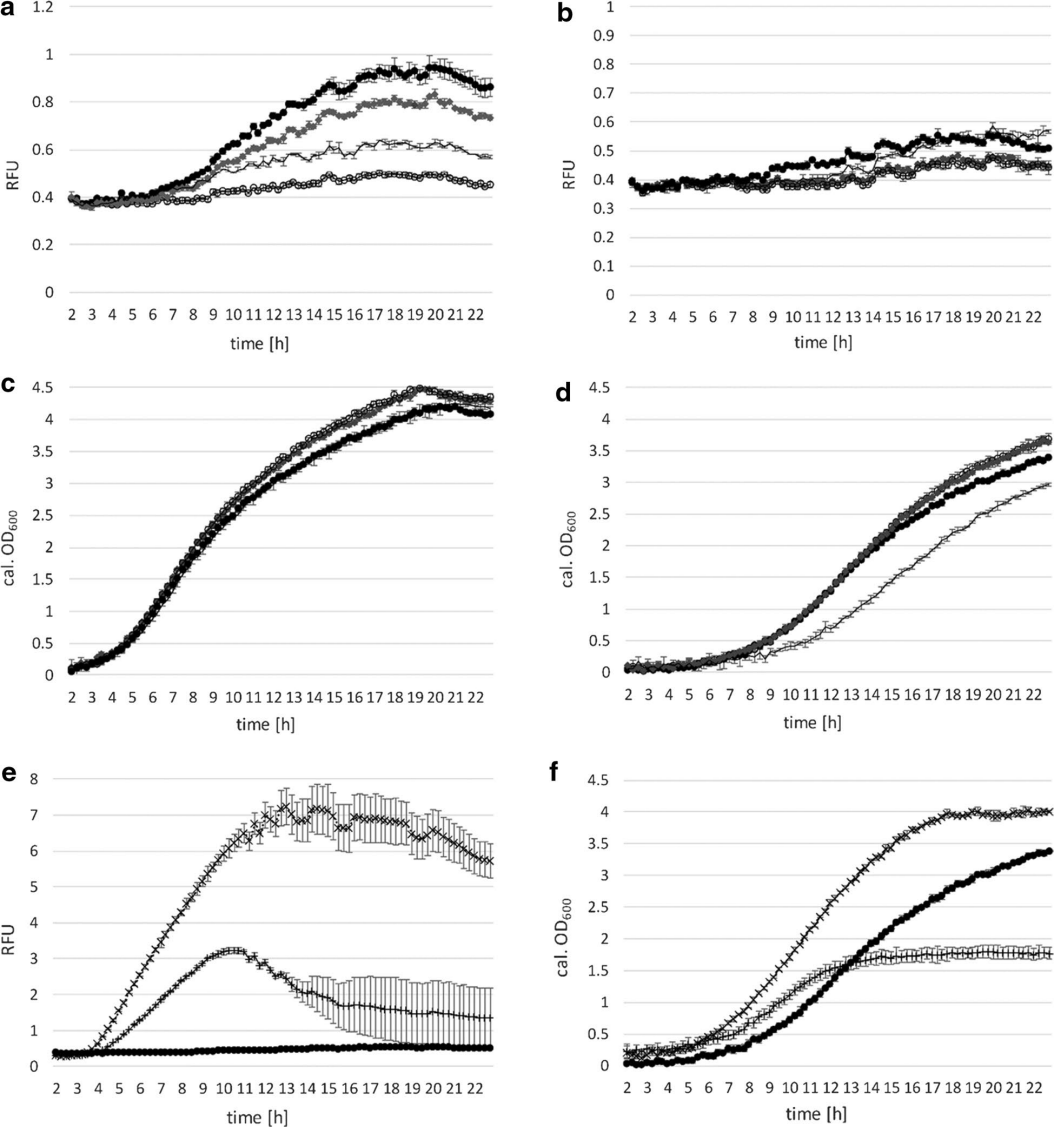
Promoter characteristics of P_lacA_ and growth behavior. **a**, **c** MRS medium with glucose and 5 μg ml^−1^ CM; **b**, **d** MRS medium with galactose and 5 μg ml^−1^ CM; **e**, **f** MRS medium with 5 μg ml^−1^ CM. Mean values of four replicates are given and standard deviations are indicated. *Filled circle* induced, 2 % lactose. *Filled diamond* induced, 0.5 % lactose. *Circle* non-induced. **a**, **b** specific expression levels of mCherry under control of P_lacA_ after induction with 0.5 and 2 % lactose (or no induction) after 2 h in BioLector^®^ micro-fermentation. Change of mCherry expression (RFUs, relative fluorescent units) over time (hours) of P_lacA_ mediated expression in comparison to the negative control is shown (*solid line*). **c**, **d** corresponding calculated OD_600_ values. **e**, **f** RFU and growth in selective MRS with lactose as main carbon source; x: induced, 2 % lactose; +: induced, 0.5 % lactose; *filled circle* induced, 2 % lactose and 2 % galactose as additional carbon source

*Lactobacillus plantarum* 3NSH harboring plasmid pCDLbu1_P_lacA__mCherry were grown on selective media either containing 2 % glucose or 2 % galactose as carbon source and were induced with 0.5 or 2 % lactose after 2 h. Induction of mCherry expression with lactose was weak, but slight increase of RFUs was observed upon the addition of 0.5 or 2 % lactose on glucose (Fig. 4a), but was not observed on galactose (Fig. 4b). Contradicting the observation by Hasan and Durr [14], we did not detect full repression in the presence of glucose.

Catabolite inhibition through diminished entry of lactose into the cell could explain why calculated OD_600_ does not increase with additional carbon source (Fig. 4c, d). Negative control (without the promoter/repressor fragment; Fig. 4a, b, solid line) grew weaker on galactose without obvious reason.

Compared to the negative control and compared to growth on glucose, minor growth impairment of the plasmid containing cells on 2 % galactose (Fig. 4d) or 2 % lactose (Fig. 4f) as carbon source was observed. Figure 4e, f show mCherry expression and growth on selective MRS medium with 0.5 and 2 % lactose as the sole carbon source and inducer. In contrast to data presented in Fig. 4a, mCherry expression increases, showing the catabolite repressive effect of glucose and galactose on P_lacA_ or on cell entry of lactose. An increase from 0.5 to 2 % lactose increases expression (Fig. 4e) and growth (Fig. 4f). However, obtained calculated OD_600_ values on galactose and induction with lactose (Fig. 4c, f, filled circle) were comparable and did not increase, albeit the twofold amount of carbon source was available.

The chromosomally encoded *lac* locus (lp_3468, lp_3469 and lp_3470) as well as existence of a second *lac* locus (lp_3483, lp_3484), as indicated for *L. plantarum* WCSF1 genomic sequence [21], might interfere with usage of lactose as inducer, since *L. plantarum* 3NSH can utilize lactose as carbon source. The expression levels of mCherry under control of endogenous P_lacA_ were rather low on glucose or galactose, but when lactose was used as sole carbon source and inducer, expression levels improved significantly.

### The synthetic promoter P_lacSynth_ is inducible by IPTG

The *lac*A promoter and the *lac*I promoter/repressor are widely used for many different *E. coli* based expression systems and many mutant versions are available [48]. Therefore, we synthesized a DNA template consisting of the promoter P_2083_ of *L. buchneri* CD034 gene LBUCD034_2083 [16], containing two operator binding sites of the *E. coli* LacI repressor. We inserted a codon optimized version of the *E. coli* LacI repressor gene (Additional file 2: Figure S2) under control of the constitutive promoter P_0234_ of the *L. buchneri* CD034 gene LBUCD034_0234 [16]. Operator binding sites (O_1_ and O_id_) for Lac repressor binding were selected according to Oehler and colleagues [30] and integrated into P_2083_. In *E. coli*, a third operator binding site (O_2_) is encoded within the coding sequence of *lac*A [31]. This downstream *cis*-acting regulative sequence is involved in DNA bending and interaction with LacI multimers. But because integration of O_2_ sequence into the mCherry coding sequence was not realizable, O_2_ was not included in our constructs.

A synthetic regulative element for mCherry expression (P_0234__*lacI*_P_2083__*mCherry*) was constructed and promoter sequence and regulative elements are shown in Fig. 1c. LacI binding sites, operator binding sites (O_1_ and O_id_), RBS and P_2083_ are indicated. The consecutive construct is termed P_lacSynth_ and cloned into pCDLbu1 (Table 2; Fig. 2a). The resulting expression vector pCDLbu1_P_lacSynth__mCherry is depicted in Fig. 2d.

**Table 2:** Plasmids and strains used in this study

Plasmid	Reference	Size (bp)	Relevant characteristics	
pET-30a	Novagen	5400	T7 promoter, T7 terminator	
pE194	[18]	3728	Erythromycin resistance gene (ermE)	
pCD256	[43]	4790	Low copy plasmid in *L. plantarum*	
pCDLbu1	[15]	5776	High copy plasmid in *L. plantarum*	
pCDLbu1_P_T7__mCherry_T_T7__Ery	This study	6425	T7 RNA polymerase specific promoter	
pCDLbu1Δ*Ec_*P_T7__mCherry_T_T7__Ery	This study	3989	T7 RNA polymerase specific promoter, without sequences for replication and selection in *E. coli*	
pCDLbu1Δ*Ec*_P_11__mCherry	[47]	3809		
pCD256_P_lacSynth__mCherry	This study	6959	Low copy plasmid; promoter P_lacSynth_; gene of interest mCherry	IPTG (1 mM)
pCD256_P_lacSynth__T7RNAP	This study	8882	Low copy plasmid; promoter P_lacSynth_; gene of interest T7 RNA polymerase	IPTG (1 mM)
pCDLbu1_P_lacSynth__mCherry	This study	8164	High copy plasmid; promoter P_lacSynth_; gene of interest mCherry	IPTG (1 mM), TMG (17 mM)
pCDLbu1_P_lacA__mCherry	This study	7917	High copy plasmid; promoter P_lacA_; gene of interest mCherry	Lactose (0.5 -2 % *w*/*v*)
pCDLbu1_P_xylA__mCherry	This study	8077	High copy plasmid; promoter P_xylA_; gene of interest mCherry	Xylose (0.2 -2 % *w*/*v*)
pCDLbu1_P_xylA(nativeRBS)__mCherry	This study	8077	High copy plasmid; promoter P_xylA_; gene of interest mCherry; native RBS and spacer sequence from *B. megaterium* DSMZ 319 *xylA*	Xylose (2 % *w*/*v*)
Strains				
*B. megaterium* DSMZ 319	DSMZ			
*E. coli* Neb10β	NEB			
*L. plantarum* CD033	[43]			
*L. plantarum* 3NSH	[17]		Plasmid cured *L. plantarum* CD033	

**Fig. 5:**
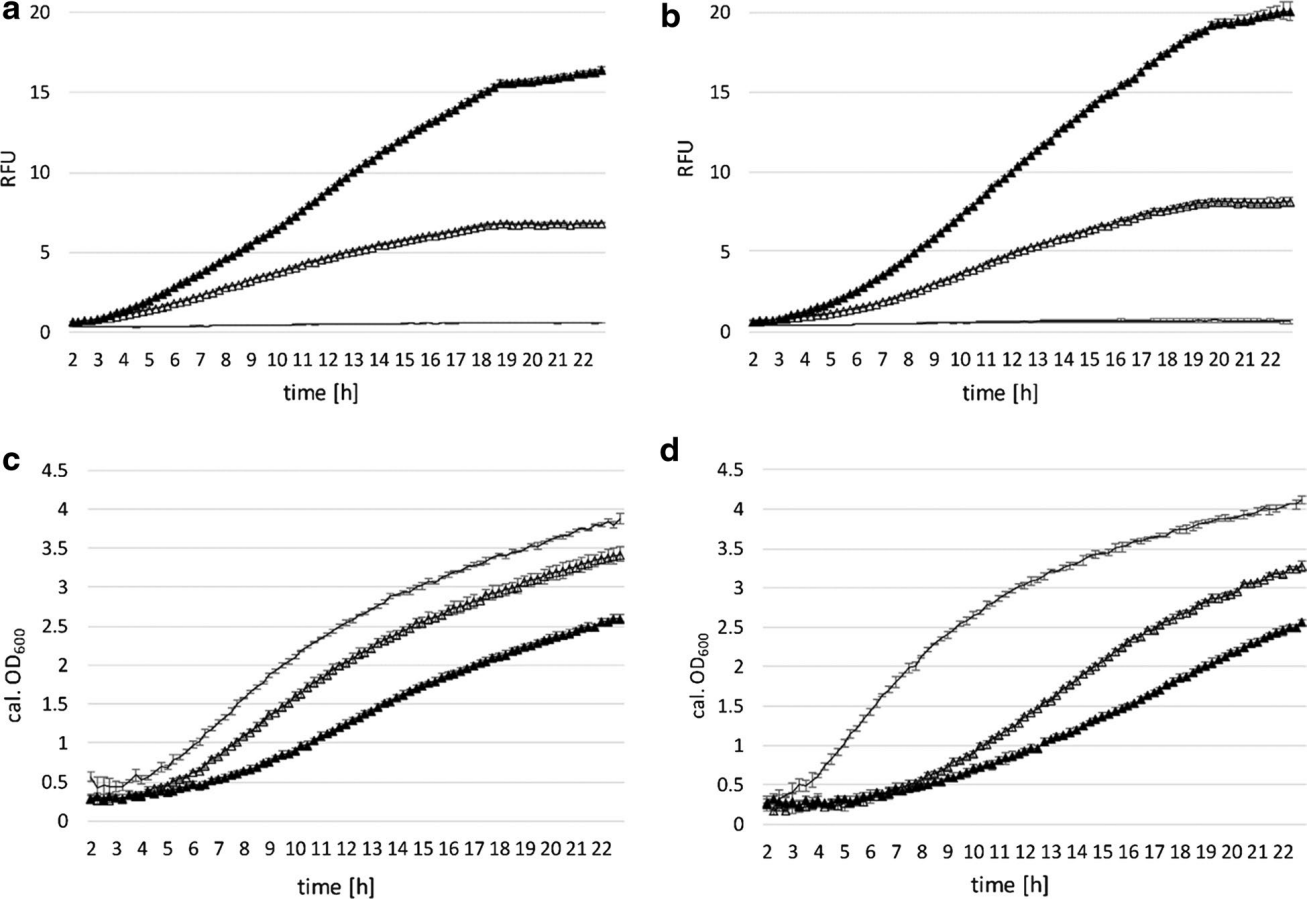
Promoter characteristics of P_lacSynth_ and growth behavior. **a**, **c** MRS medium with glucose and 5 μg ml^−1^ CM; **b**, **d** MRS medium with galactose and 5 μg ml^−1^ CM, mean values of four replicates are given and standard deviations are indicated. *Filled triangle* induced; *triangle* non-induced. **a**, **b** specific expression levels of mCherry under control of P_lacSynth_ after induction with 1 mM IPTG (or no induction) after 2 h in BioLector^®^ micro-fermentation. Change of mCherry expression (RFUs, relative fluorescent units) over time (hours) of P_lacSynth_ mediated expression in comparison to the negative control is shown (*solid line*). **c**, **d** calculated OD_600_ values. *Solid line* negative control

According to *E. colilac*-operon regulation, we tested mCherry expression subsequent to induction with IPTG. Increasing concentrations in the range of 0.1 to 5 mM (0.1, 0.5, 1.0, 2.0 and 5.0 mM) were tested and showed that already 0.1 and 0.5 mM induce P_lacSynth_ sufficiently in *L. plantarum* 3NSH. Lower IPTG concentrations (like 0.1 and 0.5 mM) are in the range of common *E. coli* implementations. Moreover, 1 mM IPTG led to saturated induction of mCherry in *L. plantarum* 3NSH (Fig. 5a, b) and no further increase of expression was observable at augmented concentrations from 1 to 5 mM (data not shown). Additionally, TMG was tested for P_lacSynth_ induction. We observed similar mCherry expression with induction of 17 mM TMG compared to 1 mM IPTG (data not shown). For *L. plantarum* NC2 it was shown that ß-galactosides are transported via ATP driven proton motive force [19]. Induction of recombinant gene expression in a fermentation setting (BioLector^®^ measurement) with IPTG (and TMG) is shown here for the first time in *L. plantarum*.

Comparative growth and induction on selective media with glucose or galactose are shown in Fig. 5c and d. Induced cultures show growth impairment (compared to the non-induced cultures) on both carbon sources, though growth on glucose as carbon source is preferred, thus leading to higher expression values and biomass (Fig. 5a, c). Overall, regarding expression levels as well as repression under non-induced conditions in both tested media variations, P_lacSynth_ performed better than P_xylA_ (Fig. 3) and P_lacA_ (Fig. 4). Negative control (without the promoter/repressor fragment) showed no mCherry expression (Fig. 5; solid line). Therefore, measured expression levels correlate to induction of P_lacSynth_ through thiogalactosides, such as IPTG (and TMG), and basal expression might be caused by weak repression of P_2083_.

Consequently, we suggest limited stoichiometric availability of the repressor LacI, resulting in incomplete repression of P_lacSynth_ by the repressor. LacI binds to the operator sites by forming tetramers, which might not be possible if LacI availability is not sufficient [31, 49]. A stronger promoter for LacI expression (instead of P_2083_) might increase repressor levels and improve transcription control. Additionally, the operator O_2_ downstream of the start codon, which is originally present within the coding sequence of β-galactosidase [31], is absent within the mCherry sequence. Therefore, bending of DNA via binding of tetrameric Lac-repressor to two adjacent operators for sufficient repression is not possible. Albeit, it was reported for *E. coli* that the presence or absence of operator O_2_ does not have an impact on *lac* operon expression anyhow [27].

Apparently, on selective medium with glucose as carbon source, the ratio of induced expression to basal expression under non-induced conditions of P_lacSynth_ was highest compared to P_xylA_ or P_lacA_ (Figs. 3, 4). However, the P_lacSynth_ mediated expression after induction is still moderate and thus appropriate for the regulation of T7 RNA polymerase based expression of mCherry. Therefore, this synthetic promoter/repressor fragment was used for establishment of the inducible T7 system in *L. plantarum* 3NSH.

### T7 RNA polymerase driven mCherry expression in *L. plantarum* 3NSH

In order to establish an orthologous expression system in *L. plantarum*, we combined the synthetic repressor/promoter system P_lacSynth_ (Fig. 1c). The adapted *E. coli* phage T7 RNA polymerase was applied to establish two compatible plasmids: one contained a codon optimized version of the T7 RNA polymerase (Additional file 3: Figure S3) under the control of P_lacSynth_ (Fig. 2f) and the second plasmid contained mCherry under control of the T7 RNA polymerase promoter P_T7_ (Fig. 2g).

**Fig. 6:**
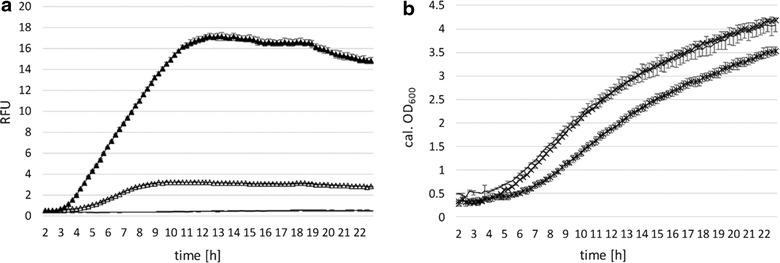
Promoter characteristics and analysis of mCherry expression under control of P_T7_, regulated by T7 RNA polymerase under control of P_lacSynth_, and growth behavior. **a** Change of mCherry expression (RFUs, relative fluorescent units) over time (hours) regulated by T7 RNA polymerase dual plasmid system in BioLector^®^ micro-fermentation is shown. T7 RNAP is under control of P_lacSynth_ and induced with 1 mM IPTG (or non-induced) after 2 h. MRS medium with glucose either 5 μg ml^−1^ CM or 5 µg ml^−1^ Ery (or both for the dual plasmid system). –x-: induced; mean values of four replicates are given and standard deviations are indicated. –: non-induced; mean values of three replicates are given and standard deviations are indicated.** b** Calculated OD_600_ values. *Solid line* negative control (n = 3; pCDLbu1Δ*Ec*_P_T7__mCherry_T_T7__Ery)

For inducing T7 RNA polymerase expression, we used 1 mM IPTG, equally to P_lacSynth_ induction (Fig. 5). Results of the expression experiment are shown in Fig. 6a. Induction with 1 mM IPTG led to augmented expression of the reporter protein compared to non-induced conditions. Some basal expression of the reporter gene was detected under non-induced conditions similarly to results with P_lacSynth_ (Fig. 5a). Therefore, the plasmid containing mCherry under control of the T7 RNA polymerase promoter (pCDLbu1Δ*Ec*_P_T7__mCherry_T_T7__Ery) was tested in absence of the second plasmid, which provides the T7 RNA polymerase (pCD256_P_lacSynth__T7RNAP). Thereby, we observed no mCherry expression neither with nor without IPTG (Fig. 6a, solid line).

Although, constitutive expression using the P_11_ promoter was significantly higher, inducible expression based on the T7 RNA polymerase system serves as a valuable tool for regulated gene expression at moderate levels. However, growth was not affected by P_lacSynth_ regulating a dual plasmid expression system (Fig. 6b) compared to strains with plasmid pCDLbu1_P_lacSynth__mCherry (Fig. 5c). This effect could also be contributed to the different plasmid backbones (pCDLbu1 and pCD256, Fig. 2d, f).

For constructing the T7 polymerase encoding plasmid, the low copy plasmid pCD256 was used (Table 2). The second plasmid (mCherry under control of P_T7_) was established from pCDLbu1 (Table 2) without *E. coli* specific sequences. Thereby we generated a smaller plasmid and less genetic load. Intentionally we introduced two different origins of replication within a cell, which is known to be preferred due to plasmid incompatibility [29]. Chromosomal integration of expression cassettes has been shown previously in *L. plantarum* [24, 36] and would be a feasible strategy for generating a stable T7 RNA polymerase expressing host strain. Such a *L. plantarum* strain would be the basis for a new T7 based expression system in a food grade host, providing specific regulation and easy exchange of any target gene that is under control of the T7 promoter P_T7_.

### Comparative analysis and semi-quantitative Western blot

The constitutive *L. plantarum* promoter P_11_ (expression vector pCDLbu1Δ*Ec*_P_11__mCherry) served as a benchmark in a comparative analysis [38, 47]. Plasmid pCDLbu1Δ*Ec*_P_11__mCherry is shown in Fig. 2e. Measurements of expression levels with plasmid pCDLbu1Δ*Ec*_P_11__mCherry were included for intrinsic comparison, because it was previously shown to yield strongest expression of mCherry amongst several tested variants in *L. plantarum* CD033 [47], the parental strain of *L. plantarum* 3NSH.

**Fig. 7:**
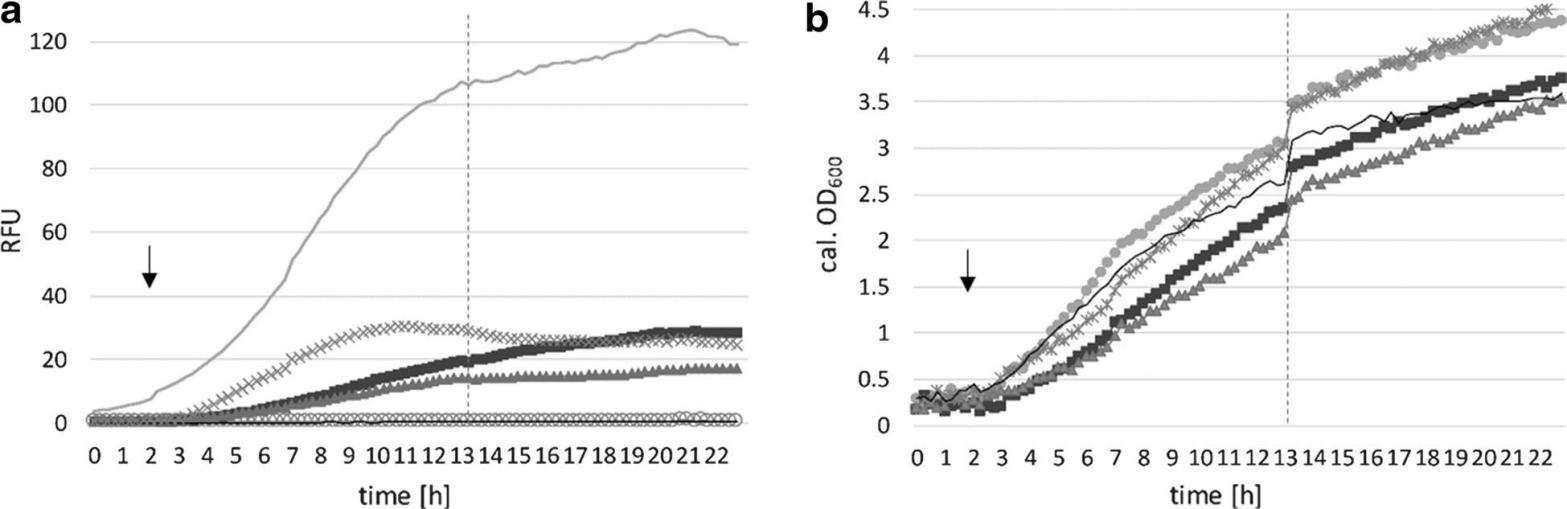
Expression and growth comparison of inducible promoters to constitutive promoter. BioLector^®^ micro-fermentation measurement for 23 h in selective MRS medium with glucose with low pH FlowerPlate at 30 °C (individual values). *Dotted vertical line* indicates sampling point (200 µl) for semi-quantitative Western blot, 13 h after start, followed by ongoing measurement, therefore OD_600_ curves are shifted after sampling. *Arrow* indicates induction time point (or absence of inducer). *Solid line* mCherry under control of constitutive P_11_ promoter (pCDLbu1Δ*Ec*_P_11__mCherry), –x-: T7 RNA polymerase based dual plasmid system induced with 1 mM IPTG, *square* P_xylA_ induced with 2 % xylose, *triangle* P_lacSynth_ induced with 1 mM IPTG, *circle* P_lacA_ induced with 2 % lactose. **a** specific expression levels of mCherry under control of inducible promoters with respective inducers. **b** corresponding calculated OD_600_ values

Expression levels of mCherry under control of P_11_ were compared to P_xylA_, P_lacA_, P_lacSynth_, and the P_lacSynth_ regulated T7 RNA polymerase, in Fig. 7a. Growth curves of producing strains and wild type are shown in Fig. 7b. MRS selective medium was used with galactose as carbon source and induction with xylose for pCDLbu1_P_xylA__mCherry, and with glucose plus induction with 2 % lactose for pCDLbu1_P_lacA__mCherry. Growth on glucose and induction with 1 mM IPTG was used for pCDLbu1_P_lacSynth__mCherry, and subsequently for the T7 dual plasmid system. P_11_ driven expression (pCDLbu1Δ*Ec*_P_11__mCherry) is more effective (Fig. 7a). Expression levels of P_lacA_ were quite low for direct comparison with promoter P_11_, but results with P_lacA_ were also included in Fig. 7a and b.

**Fig. 8:**
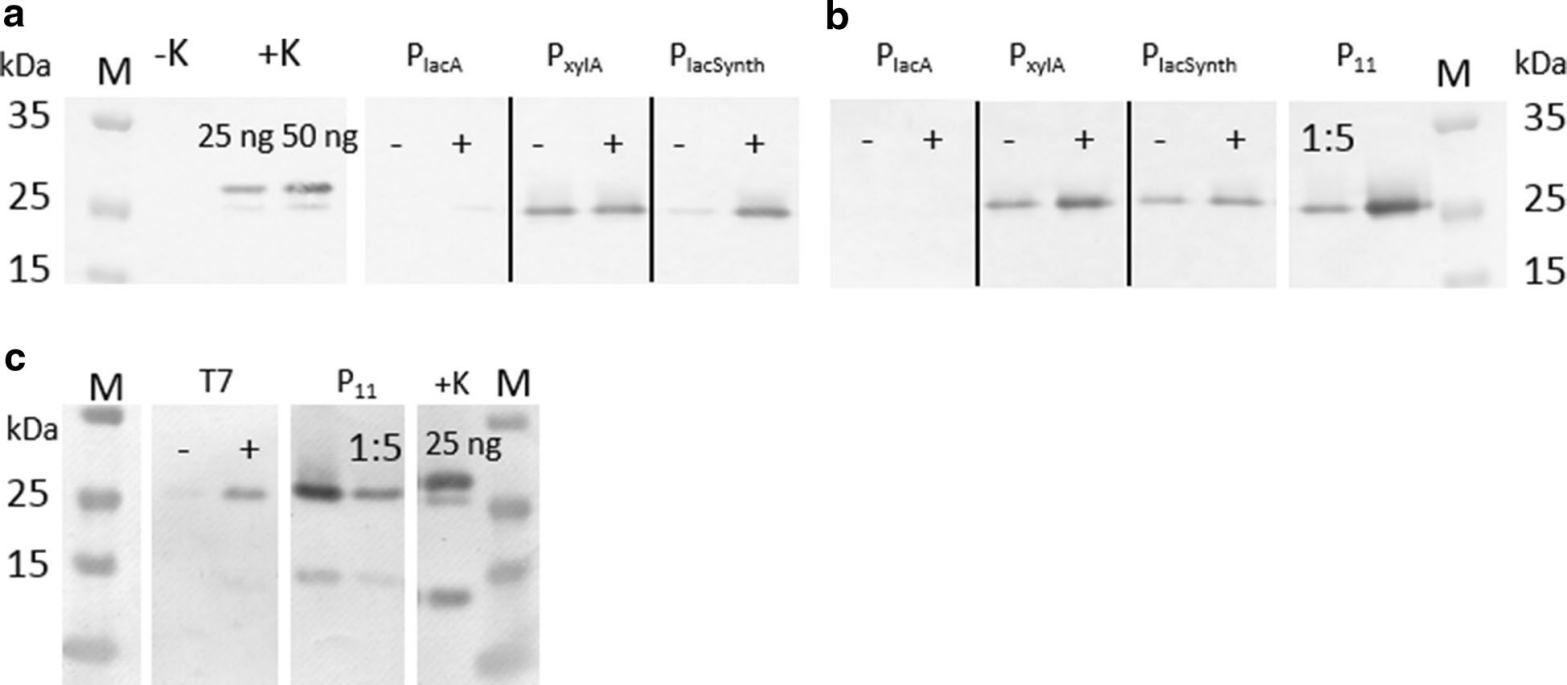
Semi-quantitative Western blot analysis. Evaluation of intracellular mCherry (26.7 kDa) expression after 13 h of BioLecture^®^ micro-fermentation measurement with low pH FlowerPlate at 30 °C. Five µg biomass per slot were applied. **a** MRS medium with glucose and 5 μg ml^−1^ CM; **b** MRS medium with galactose and 5 μg ml^−1^ CM; **c** T7 RNAP dual plasmid system, MRS medium with glucose and 5 μg ml^−1^ CM and 5 μg ml^−1^ Ery. − non-induced, + induced with the respective inducer. P_11_ sample (mCherry under control of constitutive P_11_ promoter) is additionally diluted 1:5 to adjust to expression levels of inducible promoters (in accordance BioLecture^®^ results). *M* pre-stained protein ladder; *+K* positive control mCherry(His_6_) 28.8 kDa; *−K* *L. plantarum* 3NSH wild type

A semi-quantitative Western blot of the inducible promoter systems at induced and non-induced conditions on glucose (Fig. 8a, c) or galactose (Fig. 8b) was performed. Sample point is indicated as vertical dotted line in Fig. 7 after 13 h of growth. About 5 µg biomass per slot were applied, the commercially obtained positive control (mCherry-His_6_; 28.8 kDa) was applied at concentrations of 25 and 50 ng per slot. P_11_ samples were applied undiluted and 1:5 diluted due to stronger expression compared to the inducible systems (Fig. 7a). The Western blot shows better inducibility of the P_lacSynth_ system on glucose medium (Fig. 8a) whereas P_xylA_ induction is more distinct on galactose medium (Fig. 8b) and the T7 system is inducible on glucose (Fig. 8c). We also observed basal transcription under non-induced conditions for all compared promoter/repressor systems. This is in accordance with the BioLector^®^ measurements.

Suitable and controllable expression levels were achieved by P_lacSynth_ induction with IPTG on glucose and galactose (Fig. 5a, b). Apparently, the ratio of induced expression to basal expression under non-induced conditions of P_lacSynth_ on selective medium with glucose as carbon source was best (compared to P_lacA_ or P_xylA_). Moreover, the P_lacSynth_ mediated expression after induction was still moderate and thus, more appropriate for the regulation of T7 RNA polymerase based expression of mCherry. By using a stronger promoter for *lacI*, expression, repression of P_lacSynth_ might be improved. If necessary, this strategy could also be applied for *xylR* promoter and *lacR* promoter. Best regulation of induced and non-induced conditions while yielding similar expression levels was achieved by T7 RNA polymerase dual plasmid based system induced with 1 mM IPTG (Fig. 7a, 8c). The adapted T7 RNA polymerase was successfully established here for *L. plantarum*.

## Conclusions

In this study, we tested and compared three different promoter-repressor systems for induced recombinant protein expression (red fluorescent protein mCherry) in plasmid free *L. plantarum* 3NSH. Reporter gene and regulatory elements were cloned into the high copy number plasmid pCDLbu1. The endogenous LacA promoter (P_lacA_) derived from *L. plantarum* 3NSH showed only weak reporter gene expression upon induction with 2 % lactose, which was found to be the exclusive inducer so far. Glucose and galactose acted as repressors of P_lacA_. With lactose as single carbon source better expression levels were obtained. The XylA promoter (P_xylA_) derived from *B. megaterium* DSMZ 319 was tested in combination with the expression of the repressor XylR. Upon induction with 0.2–2 % xylose, we measured increased mCherry expression during exponential phase, and repression under non-induced conditions with galactose as the carbon source. A synthetic promoter (P_lacSynth_), based on the *E. coli* derived *lac* operon resulted in moderate expression levels after induction with IPTG and TMG. P_lacSynth_ was used efficiently for the establishment of a dual plasmid system for well-regulated T7 RNA polymerase expression, and transcription of mCherry under control of the T7 RNA polymerase promoter.

Some feasible suggestions for inducible recombinant protein expression in *L. plantarum* 3NSH are presented in this study. Expression levels of recombinant protein are however, much lower as compared to expression levels driven by the constitutive P_11_ promoter. Plasmid pCDLbu1Δ*Ec*_P_11__mCherry served as a benchmark and has been described previously [47]. Additionally, differences of expression in exponential phase, initiated by varying promoters, decreased during prolonged fermentation. In stationary phase (after 22 h) mCherry levels of tested inducible promoters are aligned. Yet, depending on the recombinant protein (e.g. amino acid composition and post-translational modifications) or experiment outlook (e.g. short time setting or production of cell toxic products) promoters, which are inducible by conventional sugars or well-established inducers are of particular interest. Although general knowledge of recombinant protein expression (e.g. therapeutics or metabolites) in lactobacilli steadily increases, efficiency and expression levels are not yet comparable to *E. coli* based systems. Recombinant gene expression usually exerts additional metabolic burden for the host. This often results in unstable genetic constructs, inhibition of cell growth and/or plasmid loss. Therefore, inducible expression systems where transcription of the target gene can be tightly controlled are preferable. The presented expression systems might behave different in other *Lactobacillus* strains, the adaption of new promoter/repressor systems, and in particular a T7 RNA polymerase based expression systems for *L. plantarum,* is anticipated to contribute to a flexible genetic tool box for cell engineering and recombinant protein expression in lactic acid bacteria.

## Methods

### Enzymes and gene synthesis

All restriction and modifying enzymes, as well as Q5 DNA polymerase, were purchased from New England Biolabs (NEB). Primers (Table 1) were obtained from Integrated DNA Technologies (IDT) and phosphorylated primers were synthesized by Sigma-Aldrich.

The ribosome binding site (RBS) was identical for all constructs. Identical Shine-Dalgarno sequence (SDS) and spacer region (bold) was selected for every construct according to SOPT#9 (5´-AAGGAGG**AAATTATAC**ATG-3´), tested for efficient mCherry (start codon underlined) expression in *L. plantarum* CD033 [47].

Reporter gene mCherry and the synthetic LacR repressor/promoter fragment (P_lacSynth_) were codon optimized for *L. plantarum* WCSF1 using http://www.jcat.de/ and synthesized by GeneArt^®^ (life technologies). Promoter P_lacSynth_, T7 RNA polymerase and transcriptional terminator from *L. buchneri* CD034 D-lactate hydrogenase gene (AFS00145.1) [16] were also codon optimized as described above and synthesized by GeneArt^®^. Nucleotide sequence of the codon optimized synthetic promoter/LacI repressor system is shown in Additional file 2 and T7 RNA polymerase is shown in Additional file 3.

### Strains and cultivation conditions

Plasmids were constructed and propagated in *E. coli* Neb10β and clones were selected on LB agar plates with 100 µg ml^−1^ Ampicillin at 37 °C. Sequence positive plasmids were amplified for transformation into plasmid cured *L. plantarum* 3NSH [17]. Clones were selected on either MRS agar plates with either 5 µg ml^−1^ chloramphenicol (CM), 5 µg ml^−1^ erythromycin (Ery) or both combined at 30 °C.

In liquid medium, *E. coli* strains were cultivated under agitation at 37 °C in LB-medium. *L. plantarum* 3NSH was cultivated at 30 °C under oxygen limitation without agitation in MRS medium [7], supplemented with either 2 % (*w*/*v*) d-glucose or 2 % (*w*/*v*) D-galactose. *B. megaterium* DSMZ 319 was purchased from the “Deutsche Sammlung von Mikroorganismen und Zellkulturen” (Braunschweig, Germany) and was cultivated aerobically at 30 °C in nutrient medium. Antibiotics were added as required equally to solid media preparations.

Plasmid extraction was performed with plasmid purification kit for high-copy *E. coli* plasmids (NucleoSpin^®^ Plasmid, Macherey–Nagel). After sequence verification, plasmids were used to transform *L. plantarum* 3NSH [44]. Plasmid isolation from *L. plantarum* 3NSH was performed according to Sambrook and Russel [39] with addition of 10 mg ml^−1^ lysozyme (Merck, 105281) and RNase (R6513, Sigma) to the resuspension buffer and incubation for 30 min at 37 °C before cell lysis.

### Construction of the P_xylA_/xylR-plasmid

The *xylR* repressor/promoter fragment (P_xylA_) was amplified from genomic DNA of *B. megaterium* DSMZ 319. Therefore, an overnight culture was used for DNA extraction, with pre-treatment described for Gram-positive bacteria (DNeasy Blood and Tissue Kit, Quiagen). Primers B_mega_XylOP_out_F and B_mega_XylOP_R(*Spe*I, *Sca*I, *Bam*HI) were used for amplification of the *xylR* repressor and *xylA* promoter genes with native RBS of *xylA*. Primers mCherry_w/o_RBS_*Xba*I and p256_miniori_for were used for amplification of reporter gene mCherry and Terminator T_ldh_ from *L. casei* BL23 (L-lactate dehydrogenase gene, LCABL-06930) from pCD256_P_lacSynth__mCherry (Fig. 2h). Constructs were ligated at *Spe*I and *Xba*I complementary overhangs and amplified via a PCR using B_mega_XylOP_F_*Mfe*I, *Kpn*I and Tldh_amp_*Pst*I_R, digested with *Kpn*I and *Pst*I and cloned into the pCDLbu1 plasmid (Fig. 2a) with an origin of replication for *E. coli* and *L. plantarum* [43].

For generating expression constructs with identical RBS and spacer sequence, we exchanged the native RBS of *xylA* (Additional file 1) with the RBS sequence SOPT#9 [47]. Nucleotide sequence of final P_xylA_ is shown in Fig. 1a. Therefore, we performed a continuous PCR around the ligated plasmid with phosphorylated primers B_mega_XylOP_newRBS_*Xba*I_Phos_R and mCherry_Phos_F. After ligation, plasmid pCDLbu1_P_xylA__mCherry (Fig. 2b) was transformed into *L. plantarum* 3NSH. For screening and sequencing purposes, primers B_mega_XylOP_seq_F and B_mega_XylOP_seq_R were used.

### Construction of the P_lacA_/lacR-plasmid

The plasmid pCDLbu1_P_xylA__mCherry (Fig. 2b) was *Sac*I and *Xba*I digested and fused with the LacR repressor/promoter fragment (P_lacA_). This fragment was amplified from genomic DNA of *L. plantarum* 3NSH (DNeasy Blood and Tissue Kit, Quiagen) with the primers LacI_Lplant_F_*Sac*I and LacI_Lplant_R_*Xba*I and sequenced (sequence of P_lacR_ and P_lacA_ is shown in Fig. 1b). BLASTn analysis showed 99 % coverage (three mismatches) with the transcription regulator *lacR* gene of *L. plantarum* WCSF1. After ligation and transformation into *E. coli* Neb10β, sequence positive plasmid pCDLbu1_P_lacA__mCherry (Fig. 2c) was recovered and transformed into *L. plantarum* 3NSH. For screening and sequencing purposes, primers lacR_Gal_seq_R and lacR_Gal_seq_F were used.

### Construction of the P_lacSynth_/lacI-plasmid

Consecutive arrangement of synthetic P_lacSynth_/LacI regulon (P_0234__*lacI*_P_2083_) is shown in Fig. 1c. The promoter from *L. buchneri* CD034 gene LBUCD034_0234 [16] was selected for transcription of *lacI*, encoding the *E. coli* derived codon optimized LacI repressor (Additional file 2). The promoter from *L. buchneri* CD034 gene LBUCD034_2083 [16] was selected for regulation of the reporter gene mCherry. Operator binding sites [30] were artificially inserted into P_2083_ sequence. Operator sequence O_1_ und O_id_ were adapted from *E. coli* [31]. Both constitutive *L. buchneri* CD034 promoters were identified within our group in previous promoter library experiments (data not shown).

The synthetic promoter was amplified from the synthetic GeneArt^®^ construct with primers PlacSynth_F_*Sac*I_*Eco*RI and M13_R_*Nhe*I, digested with *EcoRI* and *Bam*HI and ligated into *Eco*RI and *Bam*HI digested pCD256 [43], receiving pCD256_P_lacSynth_. This plasmid was amplified and proliferated in *E. coli*. Reporter gene mCherry was amplified from pCDLbu1Δ*Ec_*P_11__mCherry [47] with primers mCherry_RBS_*Xba*I and mCherry_R_*Bam*HI, digested and ligated with pCD256_P_lacSynth_ (cut *Xba*I and *Bam*HI) plasmid. After transformation of *E. coli* and positive colony screening, pCD256_P_lacSynth__mCherry (Fig. 2h) was recovered and the insert was amplified via PCR with primers PlacSynth_F_*Sac*I_*Eco*RI and Tldh_amp_R_*Pst*I, followed by digestion with *Eco*RI and *Pst*I and ligation into digested pCDLbu1 (Fig. 2a) vector. The plasmid pCDLbu1_P_lacSynth__mCherry (Fig. 2d) was amplified in *E. coli* and subsequently introduced into *L. plantarum* 3NSH. For screening and sequencing purposes, primers mCherry_seq_R, mCherry_seq_F and Cat_seq 2_back were used.

### Construction of the T7 RNA polymerase based dual plasmid expression system

DNA was amplified with the primers T7_RNAP_Lp_RBS and T7_RNAP_Lp_Term_R_*Sal*I from a synthetic template. The fragment was *Xba*I and *Sal*I digested and ligated into the *Xba*I and *Sal*I digested pCD256_P_lacSynth__mCherry plasmid (Fig. 2h), thus receiving the plasmid pCD256_P_lacSynth__T7RNAP (Fig. 2f).

The second plasmid (pCDLbu1Δ*Ec*_P_T7__mCherry_T_T7__Ery, Fig. 2g) was cloned stepwise. The reporter gene mCherry was amplified with primer M13_2_F and mCherry_R_*Bam*HI from plasmid pCDLbu1_P_lacSynth__mCherry (Fig. 2d), digested with *Xba*I and *Bam*HI and ligated into digested pET-30a plasmid (Table 2). Primers T7-Promoter_*Sac*I and T7-Terminator_*Sal*I were used to amplify the P_T7__mCherry_T_T7_ fragment from the established pET30a-mCherry plasmid. The erythromycin resistance gene (*ermE*) was amplified with primers ery_*Kas*I_back and oripE194_seq_back from pE194 [18]. The *ermE* fragment was digested with *Cla*I to fuse it with the *Cla*I digested P_T7__mCherry_T_T7_ fragment, followed by an enrichment PCR with primers ery_*Kas*I_back and T7-Terminator_*Sal*I. The resulting fragment was digested with *Kas*I and *Bsp*EI and ligated into the *Kas*I and *Xma*I digested plasmid pCDLbu1 [15], resulting in the plasmid pCDLbu1_P_T7__mCherry_T_T7__Ery. After amplification of the plasmid in *E. coli* JM109, *E. coli* specific sequences (pMB1 origin of replication and ampicillin resistance gene) were removed by PCR with primers Ery_F_*Nhe*I and M13_R_*Nhe*I. The PCR product of plasmid pCDLbu1Δ*Ec*_P_T7__mCherry_T_T7__Ery (Fig. 2g) was digested with *Nhe*I, circularized by ligation and directly used to transform *L. plantarum* 3NSH [44].

Subsequent to sequence verification of a colony harboring plasmid pCDLbu1Δ*Ec_*P_T7__mCherry_T_T7__Ery was used for establishing competent cells and transformed with plasmid pCD256_P_lacSynth__T7RNAP, resulting in a strain carrying two different expression vectors. After transformation, cells were selected on MRS plates with 5 µg ml^−1^ CM and 5 µg ml^−1^ Ery. Colonies were screened for both expression plasmids verified by sequencing.

### Construction of negative controls

For the inducible promoter/repressor constructs, a negative control plasmid was established by removing the whole inserted promoter/repressor fragment (plasmid with mCherry coding sequence and terminator; termed empty). The plasmid backbone, which is identical for every construct, was amplified with primers P_empty__*Sac*I_R and mCherry_RBS_*Sac*I_F from pCDLbu1_P_lacSynth__mCherry. The thereby established plasmid pCDLbu1_X_mCherry allows testing for mCherry expression, driven by possible read through from upstream regulatory sequences or possible unknown upstream promoter sequences.

As negative control for the T7 RNA polymerase dual-plasmid system, we used a clone harboring only plasmid pCDLbu1_P_T7__mCherry_T_T7__Ery. Thereby we tested if any other factors except T7 RNA polymerase contributes to mCherry expression.

### Induction conditions

Over-night cultures were adjusted to OD_600_ 0.2 in the respective liquid medium. After 2 h of growth at 30 °C in the BioLector^®^ micro-fermentation system, cultures were induced with the respective inducer 1:10 into each well, thus requiring that preparations of each inducer is tenfold concentrated in MRS-medium. Non-induced cells were prepared and tested simultaneously, but without the inducer (MRS medium only).

Tested sugars were used in the D(+)-configuration and weighted as solids (weight) per volume medium (*w*/*v*). The promoter P_xylA_ was induced with xylose. Either 0.2, 1 or 2 % xylose were used for induction. Therefore, 100 or 200 g l^−1^ D-xylose was added to the medium (MRS 5 µg ml^−1^ CM without glucose), heated in a water bath and sterile filtrated (0.2 µm) and diluted accordingly. Other preliminary tested sugars such as fructose, arabinose, maltose, as well as galactose were prepared likewise.

The synthetic promoter P_lacSynth_ was induced with IPTG (VWR) and TMG (M8146, Sigma). Standard final concentration for IPTG was 1 mM. Therefore, 10 mM IPTG was dissolved in selective MRS medium and 1:10 diluted into respective wells. For testing minimum and maximum induction concentrations, we used dilutions ranging from 0.1 to 5 mM IPTG per well (0.1, 0.5, 1, 2 and 5 mM). 17 mM TMG was also tested for induction of P_lacSynth_ in selective MRS medium, as well as 2 % lactose. For P_lacA_ standard conditions were selective MRS medium with 2 % glucose, 2 % maltose or 2 % galactose or without additional carbon source and induction with 0.5 or 2 % lactose after 2 h.

### BioLector^®^ and Tecan reader measurements of intracellular mCherry expression

Pre-measurements were performed in an Infinite^®^ M1000 PRO Tecan microplate reader as described elsewhere [47]. The BioLector^®^ micro-fermentation system (m2p-labs Germany) was also used for online measurement. Overnight cultures (in selective MRS medium with glucose or galactose) were diluted to an OD_600_ of 0.2 in the respective liquid medium. 720 μl of each sample were pipetted per well of MTP-48-BOH FlowerPlate^®^ (low pH, Lot No. 1408) or MTP-48-B FlowerPlate^®^ (without optodes, Lot No. 1402) in quadruplicates and sealed with sterile tape adhesive sealing (Nunc, 732-2610). Samples were induced after 2 h of growth. Under sterile conditions 80 µl of the particular inducer (tenfold concentrated in MRS medium) was pipetted into the respective well. 80 µl MRS medium were added to non-induced samples and controls, and plates were covered again with sealing tape. Results were analyzed after 23 h using the BioLection 2.3.13 software using a previously described calibration curve for *L. plantarum* [47]. Calibration parameters were set for 30 °C according to the manufacturer’s recommendations.

### SDS-PAGE and Western blot analysis

For Western blot analysis of intracellular mCherry (to compare the expression levels of under induced and non-induced conditions in selective MRS medium with glucose as carbon source) cells were collected at late exponential phase after 13 h of growth (dotted vertical line in Fig. 7). Recombinant purified mCherry with His_6_-tag (28.8 kDa) was purchased from BioVision (4993-100) and used as a positive control in defined concentrations per slot (25 and/or 50 ng). Per slot we applied samples corresponding to 5 µg biomass each (calculated as described below). The reference strain (pCDLbu1Δ*Ec*_P_11__mCherry) was applied undiluted and 1:5 diluted for adaption to mCherry yields obtained by induction of the inducible promoters.

The pellet of 200 µl culture was washed with PBS, centrifuged and pellet was re-suspended in 200 µl PBS. OD_600_ was measured of each sample. For analyzing equal amount of biomass 0.4/OD_600_ for each sample was calculated and used for intracellular analysis. A spatula tip of zirconium beads (BMBZ 100-250-17) was added to each sample, followed by alternating 30 s vortex and 30 s on ice; repeated for ten times. To remove cell debris and beads, samples were centrifuged at 4 °C full speed and supernatant was transferred into a fresh tube. A volume of 15 µl of each sample were mixed with 2 × LDS loading buffer and incubated at 99 °C for 10 min. Afterwards, 15 µl per sample and 5 µl protein ladder (Fermentas, SM0671) were loaded onto a NuPAGE^®^ 12 % BisTris gels and electrophoresis was run with MOPS buffer. The gel was blotted onto a PVDF membrane. Anti mCherry antibody (Biovision, 5993-100; 1:10.000) and AP-linked anti-rabbit secondary antibody (Sigma A9919; 1:20.000) were used for detection of mCherry. BCIP/NBT Color Development Substrate (Promega, S3771) was used for staining the blot.

## Additional files

10.1186/s12934-016-0448-0 Comparison of RBS and spacer sequence of P_xylA (native RBS)_ and P_xylA_. 
10.1186/s12934-016-0448-0 Sequence of the codon optimized version of the *E. coli* *lacI* repressor gene and P*lacSynth*.
10.1186/s12934-016-0448-0 Sequence of the codon optimized version of the T7 RNA polymerase gene.
