# Supplementary figures and images for: Evaluation of novel inducible promoter/repressor systems for recombinant protein expression in Lactobacillus plantarum
Source: Microb Cell Fact. 2016 Mar 10;15:50. doi: 10.1186/s12934-016-0448-0 (PMC4785742; doi:10.1186/s12934-016-0448-0)

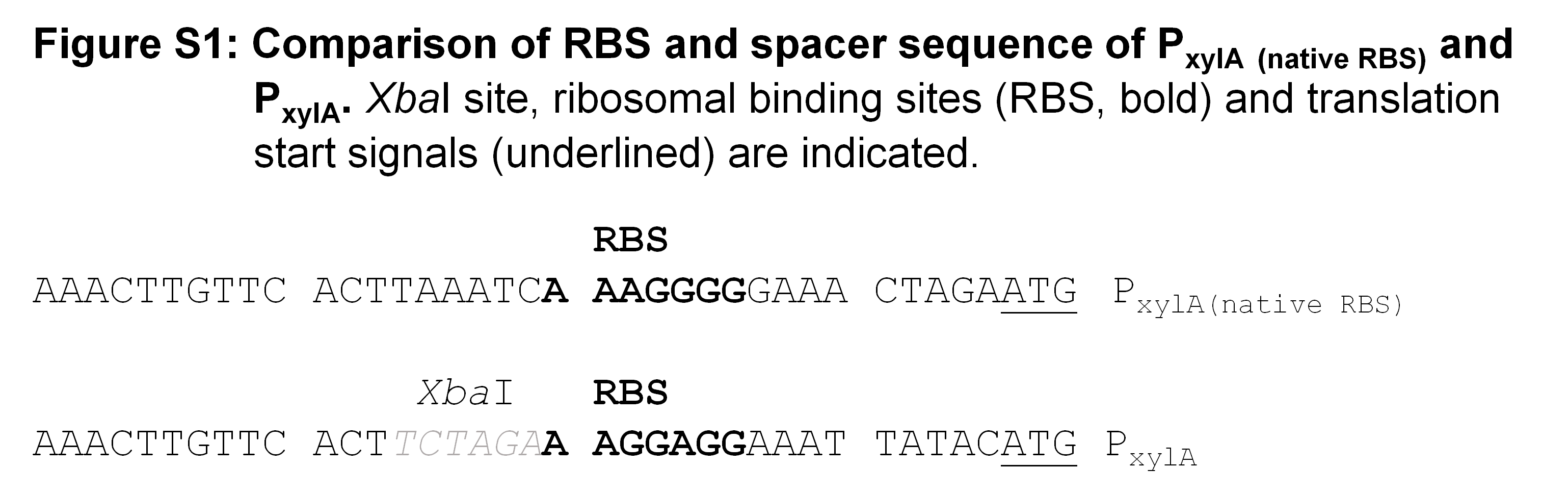

Supplement: Supplementary file 1 — 10.1186/s12934-016-0448-0 Comparison of RBS and spacer sequence of PxylA (native RBS) and PxylA. [file 12934_2016_448_MOESM1_ESM.tif]
